# Nicotinic receptors promote susceptibility to social stress in female mice linked with neuroadaptations within VTA dopamine neurons

**DOI:** 10.1038/s41386-022-01314-4

**Published:** 2022-04-22

**Authors:** Vanesa Ortiz, Renan Costa Campos, Hugo Fofo, Sebastian P. Fernandez, Jacques Barik

**Affiliations:** 1 Université Côte d’Azur, Nice, France; 2 Institut de Pharmacologie Moléculaire & Cellulaire, CNRS UMR7275 Valbonne, France

**Keywords:** Stress and resilience, Reward

## Abstract

There are about twice as many women as men who experience depression during their lifetime. Although life circumstances and especially exposure to stressful situations constitute a major risk factor to develop depression, the underlying mechanisms have yet to be unraveled. We employed the chronic social defeat procedure to elicit depressive-like symptoms in females and ketamine to validate the model. We performed ex-vivo patch clamp recordings to assess cellular adaptations and used pharmacological agents to dissect these deregulations. Chronic social defeat exposure triggers a hyperactivity of VTA putative dopamine (DA) neurons in females susceptible to stress but not resilient ones. This hyperactivity was fully reversed by a single administration of ketamine. In virally-identified brain circuits of both susceptible and resilient females, we found a hypercholinergic tone to the VTA arising from the laterodorsal tegmentum. Application of puffs of nicotine revealed a decreased sensitivity of DA neurons in resilient mice when compared to naive or susceptible ones. The in vivo acute administration of the positive allosteric modulator for α7 nicotinic acetylcholine receptors (nAChRs) not only increased susceptibility to stress by enhancing activity of VTA DA neurons, but also triggered a switch in phenotype from resilient to susceptible. Our data unravel dysregulations of VTA DA neurons activity exclusively in females exhibiting depressive-like symptoms and identify VTA nAChRs as key molecular substrates that exacerbate susceptibility to stress.

## Introduction

Depression is a major human blight causing excessive disability and mortality, and hence constitutes an overall global economic and social burden. The World Health Organization (WHO) estimates that depression affects more than 350 million people worldwide and that number of cases will increase over the years (WHO, 2017). Up to 30% of patients suffering from depression are resistant to current treatments [1]. Importantly, there is a two-fold preponderance of depression in women and this ratio persists within their lifetime [2]. Despite this obvious gender-gap, most research has been mostly performed using male subjects [3]. This hindered the development of effective treatment to cure, or at least diminish, depressive symptoms in females. Although mood changes occur following hormonal fluctuations [4, 5], inherited traits and adverse life experiences constitute major risk factors of depression [2, 6]. Hence, there is a recent surge in the development, or adaptations, of preclinical models to best mimic behavioral symptoms reminiscent of depression in females [3]. Amongst these models, the chronic social defeat stress procedure has been extensively employed to decipher the neurobiological mechanisms underpinning susceptibility and resilience to depression in males [7, 8]. Recent modifications to this protocol include pharmacogenetic-guided aggression towards females, the induction of aggressive behavior in females by housing them with male mice, or the application of male odorants to females paired with exposure to a dominant male [9–12]. Adapted to females, the chronic social defeat efficiently induced two behavioral hallmarks of depression, social avoidance and anhedonia, as well as sustained increases in anxious states [9, 10, 13]. Like it has been described in males, a subset of female mice displayed resiliency to chronic social defeat as evidenced by normal levels of social interaction and hedonic responses [9, 10]. Nevertheless, the underlying cellular mechanisms driving susceptibility and resilience to chronic social defeat in females have yet to be determined.

Over the last decade, it has been well-documented in both humans and rodents that marked dysregulation of the reward system were associated with depressive-like symptoms [7]. Male mice which are exposed to chronic social defeat develop social avoidance and anhedonia and exhibit a robust and sustained increase in the activity of dopamine (DA) neurons from the ventral tegmental area (VTA) [14–18]. These cellular maladaptations reflect a susceptibility to stress and are causally associated with the appearance of depressive-like symptoms [15, 16]. In contrast, male mice resilient to chronic social defeat fail to show abnormal behaviors and lack dysregulation of VTA DA neurons [17, 18].

Several molecular determinants of VTA adaptations have been identified and therefore enhance our understanding of the mechanisms underpinning depression in males [19]. Firing of VTA DA neurons is tightly controlled by multitudinous systems and inputs from other brain structures [20, 21]. Among those, cholinergic inputs from pontine areas have been shown to encroach and promote bursting activity of this cell population [22, 23]. Acetylcholine modulation of dopaminergic firing involves muscarinic and nicotinic acetylcholine receptors (nAChRs), the latter receiving much attention due to its implication in nicotine’s rewarding effects and tobacco addiction [24]. A wide diversity of nAChRs are expressed in the VTA in somatic, dendritic and presynaptic localizations, giving ample opportunity for a modulatory role [25]. In particular, α7 homomeric nAChRs have recently been shown to contribute to depressive-like behaviors and dopaminergic mal-adaptations induced by exposure to repeated social defeat in male mice [26]. It is however presently unknown whether a role for this cholinergic/nicotinic system participates in the pathophysiology of depressive-states in the female population.

Hence in this study, through pharmacological, viral tracing and electrophysiological approaches, we dissected the cellular dysregulation occurring in VTA DA neurons following chronic social defeat in females. We uncovered that susceptibility and resilience to social stress in females are associated with distinct maladaptive cellular responses of VTA DA neurons and are dependent on different sensitivity of nicotinic systems.

## Methods and materials

### Animals

Adult female C57BL/6 J mice (8 weeks; Janvier Labs, France) were used as experimental subjects. Mice were group housed (5 per cage), and habituated to housing conditions and experimenter handling for 1 week before starting the social defeat stress procedure. Adult male CD1 mice (former breeder aged > 4 months, Janvier Labs, France) were used as aggressors. CD1 mice were housed alone and screened during 3 days to assess their aggressive behavior towards female mice (latency to attack less than 1 min). Heterozygous female choline acetyltransferase ChAT-IRES-Cre Knock-In mice (ChAT-Cre mice, The Jackson Laboratory, stock number: 006410) were backcrossed for 9 generations on a C57BL/6 J and used to specifically target cholinergic neurons. All mice were maintained in a 12 h–12 h light-dark cycle at a room temperature of 21–22 °C, with food and water available ad libitum. All behavioral tests took place during the light cycle, with exception of the sucrose preference test (see below). All procedures were in accordance with the recommendations of the European Commission (2010/63/EU) for care and use of laboratory animals, and approved by the French National Ethical Committee. The number of mice used for each behavioral and electrophysiological experiments can be found in the Supplementary Table.

### Urine collection and application

To collect urine, we followed the method described previously [27]. The urine samples were applied immediately after the collection or stored at 4 °C for 24 h. We obtained urine from a new male CD1 mouse every day. These mice were not used as resident CD1 aggressors. Cotton swabs were employed to apply urine around the vaginal orifice, tail and lower back of females.

### Social defeat stress procedures

The chronic social defeat procedure was performed as previously described [10]. Female mice were impregnated with urine of male CD1 mice and immediately after, were placed in a resident CD1 home cage. Following the defeat session, experimental mice were maintained in sensory, but not physical, contacts with the male CD1 through a semi-permeable barrier. This procedure was repeated for 10 consecutive days. Each female subject faced a new CD1 every day and defeat sessions were limited to a maximum of 5 min. Undefeated mice (Naive) were not confronted with a dominant male and were not impregnated with urine but lived in similar housing conditions, separated by a semipermeable barrier.

The subthreshold social defeat procedure was modified from previous work [26]. Female mice were subjected to a defeat session for 2 min, followed by 20 min in sensory contacts with the aggressor CD1 mouse through a semipermeable barrier. Immediately after, this procedure was repeated once, and females were then re-grouped in their home cages. One day later, behavioral testing was performed.

### Social interaction

One day after the last defeat (day 11 or day 2 for the chronic social defeat and subthreshold social defeat procedures respectively), the social interaction (SI) test was performed in a low luminosity environment (7 ± 2 lux). Experimental mice were exposed to two consecutive sessions (180 s each) in an open-field containing initially an empty perforated box (No target “NTg” condition), which was then replaced by a box containing an unfamiliar CD1 mouse (Target “Tg” condition). The time spent in the interaction zone surrounding the box was recorded and used as an index of social interaction. We used ANY-maze for videotracking. The performance of each mouse was expressed as ratio: [time spent in the interaction zone during Tg / time spent in the interaction zone during NTg], with a ratio < 1 as index of susceptibility and a ratio > 1 as an index of resilience.

### Drugs

PNU-120596 (Tocris Cookson, Bristol, UK) was dissolved in 5% DMSO in saline and administered (1 mg/kg, i.p) 15 min before the subthreshold social defeat procedure or SI test. Ketamine (Centravet, France) was diluted in saline (0.9% NaCl) and given intraperitoneally (20 mg/kg) 24 h previous the SI test. Nicotine (Sigma, France) was diluted in artificial cerebrospinal fluid (aCSF) for patch recordings (see below).

### Elevated zero maze

Twenty-four hours after the social interaction test, mice were tested in the elevated zero maze (O-Maze) in order to evaluate their anxiety levels. The O-maze consisted of two open arms and two closed arms alternating in quadrants (width of walking lane: 5 cm, total diameter: 55 cm, height of the walls: 12 cm, elevation above the floor: 60 cm). The testing room was dimly lit (12 ± 2 lux in the open arms) and each single session lasted 5 min. The percentage of time spent in the open arms ([time spent in the open arms / (time spent in open arms + time spent in closed arms)] × 100) was calculated for each mouse.

### Sucrose preference

Sucrose preference was measured in a two-bottles choice procedure. Mice were acclimatized to two bottles containing water for 48 h before sucrose preference test. Following the O-Maze, mice were allowed to freely drink from a bottle of water or sucrose (0.5%) during 12 h (overnight). The bottles were weighed and sucrose preference was calculated as follow [volume of sucrose consumed/ (volume of sucrose + volume of water consumed)] × 100.

### Estrous cycle assessment

The estrous cycle was monitored visually and histologically using the vaginal lavage method as previously described [28, 29]. Vaginal samples were collected immediately before the electrophysiological recordings. For mice employed in subthreshold social defeat experiments, the estrous cycle was determined by visual inspection [28].

### Stereotaxic injections

Stereotaxic viral injections were performed as previously described [30] using a stereotaxic frame (Kopf Instruments) under general xylasine and ketamine (10 mg/kg and 150 mg/kg respectively, Centravet-France) anaesthesia. To identify cholinergic neurons in the laterodorsal tegmentum (LDTg) nucleus projecting to the VTA, female ChAT-Cre mice (5 weeks old) were injected with retro-AAV-CAG-FLEX-tdTomato bilaterally in the VTA (AP: − 3.2 mm, ML: ± 0.6 mm, DV: − 4.7 mm from the bregma adjusted from the mouse brain atlas [31]). The injection rate was set at 100 nl/min. Experiments started 3 weeks after injections to allow sufficient viral expression and recovery from the surgery. Correct injection sites were verified for each mouse and only animals with adequate bilateral injection sites were considered for statistical analyses.

### Ex vivo patch-clamp recordings

Recordings were performed as described previously [30]. Mice were anesthetized (Ketamine 150 mg/kg / Xylazine 10 mg/kg) and transcardially perfused with cold aCSF for slice preparation on day 11–14 of the chronic social defeat procedure. For VTA recordings, horizontal 250 μm slices were obtained in bubbled ice-cold 95% O_2_, /5% CO_2_ aCSF containing (in mM): KCl 2.5, NaH_2_PO_4_ 1.25, MgSO_4_ 10, CaCl_2_ 0.5, glucose 11, sucrose 234, NaHCO_3_ 26. Slices were then incubated in aCSF containing (in mM): NaCl 119, KCl 2.5, NaH_2_PO_4_ 1.25, MgSO_4_ 1.3, CaCl_2_ 2.5, NaHCO_3_ 26, glucose 11, at 37 °C for 1 h, and then kept at room temperature. Both spontaneously and non-spontaneously active putative VTA DA neurons were recorded. For LDTg recordings, coronal slices (250 μm) were obtained using the same solutions but recovery at 37 °C lasted 15 min.

Slices were transferred and kept at 31–32 °C in a recording chamber superfused with 2.5 ml/min aCSF. Visualized whole-cell voltage-clamp or current-clamp recording techniques were used to measure spontaneous synaptic activity or excitability, respectively, using an upright microscope (Olympus France). Putative DA neurons were identified by anatomical location and electrophysiological profile, as previously described [30]. Recordings were perfomed in the lateral VTA, in neurons medial to the medial terminal nucleus of the accessory optic tract. Current-clamp experiments were obtained using a Multiclamp 700B (Molecular Devices, Sunnyvale, CA). Signals were collected and stored using a Digidata 1440 A converter and pCLAMP 10.2 software (Molecular Devices, CA). In all cases, access resistance was monitored by a step of −10 mV (0.1 Hz) and experiments were discarded if the access resistance increased more than 20%. Internal solution contained (in mM): K-D-gluconate 135, NaCl 5, MgCl_2_ 2, HEPES 10, EGTA 0.5, MgATP 2, NaGTP 0.4. Depolarizing (0–300 pA) or hyperpolarizing (0- −450 pA) 800 ms current steps were used to assess excitability and membrane properties of LDTg and VTA neurons. For recording spontaneous excitatory post-synaptic currents (sEPSCs), cells were voltage clamped at −70 mV and recorded for a period of 5 min in the presence of picrotoxin 50 μM. All electrophysiological data was analysed off-line using Clampfit v11.2 (Molecular Devices, UK). Current clamp experiments were used to determine passive membrane properties and excitability profile as described [32]. sEPSC were detected using threshold detection with the following parameters to define events (amplitude>5 pA; duration 1–6 ms); events were visually inspected by experienced experimenters. A minimum of 50 events were isolated per cell.

For electrophysiology recordings of DA cells in response to nicotine application, nicotine was applied by a ‘puff’ with pressure pulses controlled by a Picospritzer III (General Valve). A drug-filled pipette was moved within 20–40 μm from the recorded neuron and a pClamp protocol triggered 5 sweeps of nicotine (10 mM) puff applications onto the recorded neuron. Puffs with 20 psi ejection pressure and 20–100 ms duration were interspersed by 70 s. The average peak amplitudes of the inward currents in response to acute nicotine puff were then compared between groups. To test the impact of PNU-120596 onto nicotine-evoked responses, VTA brain slices were incubated for 10 min with either 1 μM PNU-120596 dissolved into aCSF (containing 0,1% DMSO) or aCSF (containing vehicle alone). Nicotine was puffed onto VTA DA neurons and data analyzed as described above.

### Data analysis

D’Agostino-Pearson’s and Bartlett’s tests were used to determine the normality of the distributions and homogeneity of variance, respectively. Normal data were analyzed by Student’s t-test or one-way ANOVA, with repeated measures when required, followed by Tukey’s multiple comparison post hoc test. For data without normal distribution or homogeneity of variance, we employed Kruskal-Wallis’ test followed by Dunn’s multiple comparison test. Results are expressed as the mean ± the standard error of the mean (SEM) or the median and interquartile range, and were analyzed using GraphPad Prism 8. sEPSCs were depicted also as frequency distribution histograms. Statistical significance was set at *p* < 0.05.

## Results

### Chronic social defeat triggers depressive-like behaviors in female mice reversed by ketamine

**Fig. 1:**
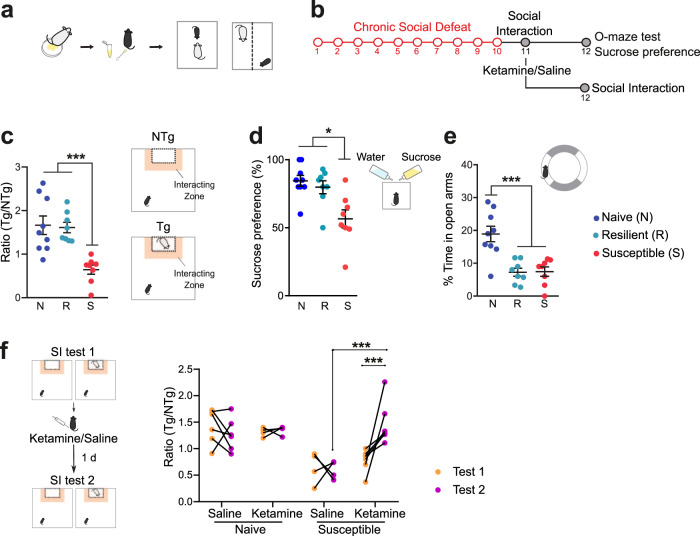
Chronic social defeat promotes depressive-like behaviors in female mice. **a** Schematic of the social defeat procedure. **b** Experimental timeline. **c** Susceptible mice displayed social avoidance, while resilient females showed similar social interaction ratio than naive mice. **d** Sucrose preference was decreased in susceptible female mice. **e** Defeated females exhibited increased anxiety responses in the O-Maze test. (n/group: Naive = 9; Resilient = 8; Susceptible = 8) **f** Naive and susceptible females were injected with saline or ketamine (i.p, 20 mg/kg) after the SI test on day 11, and the social exploration was re-tested 24 h following drug injection. Ketamine reversed the social avoidance in susceptible females, without changes in the performance of naive mice. (n/group: Naive-Saline = 6; Naive-Ketamine = 4; Susceptible-Saline = 4; Susceptible-Ketamine = 7). All data are expressed as the mean ± SEM.

Following 10 days of social defeat in female mice (Fig. 1a, b), we screened for the appearance of depressive-like behaviors and anxious states. We found that approximately 50% of defeated females displayed social avoidance and anhedonia, evidenced by decreased social interaction (Fig. 1c, One-way ANOVA, F (2, 22) = 12.7, *p* = 0.0002; Tukey’s comparisons test: ***p < 0.001) and reduced sucrose preference (Fig. 1d, One-way ANOVA, F (2, 22) = 8.26, *p* = 0.0021; Tukey’s comparisons test: ***p < 0.001). The other 50% of defeated females did not exhibit a depressive-like phenotype and maintained control levels of social exploration and sucrose preference (Fig. 1c, d). This indicates that half of the female tested showed a susceptible phenotype, whereas the others were resilient to chronic social defeat. In contrast, all defeated females showed reduced time in the open arms in the O-maze test, indicative of a generalized increased in anxiety levels (Fig. 1e, One-way ANOVA, F (2, 22) = 14.1, *p* = 0.0001; Tukey’s comparisons test: ***p < 0.001). To test the therapeutic efficacy of ketamine on depressive-like behaviors following chronic social defeat, females that showed a susceptible phenotype in the social interaction (Test 1), were injected with an acute i.p. injection of saline or ketamine (20 mg/kg), and submitted to a second social interaction test 24 h after (Test 2). Social aversion was still present in saline-treated mice, but ketamine injection completely rescued the phenotype (Fig. 1f, Repeated-measures two-way ANOVA: F (3,17) = 4.52, *p* = 0.017; Tukey’s comparisons test: ***p < 0.0001).

### Susceptibility to chronic social defeat is associated with cellular adaptations in VTA DA neurons of females

**Fig. 2:**
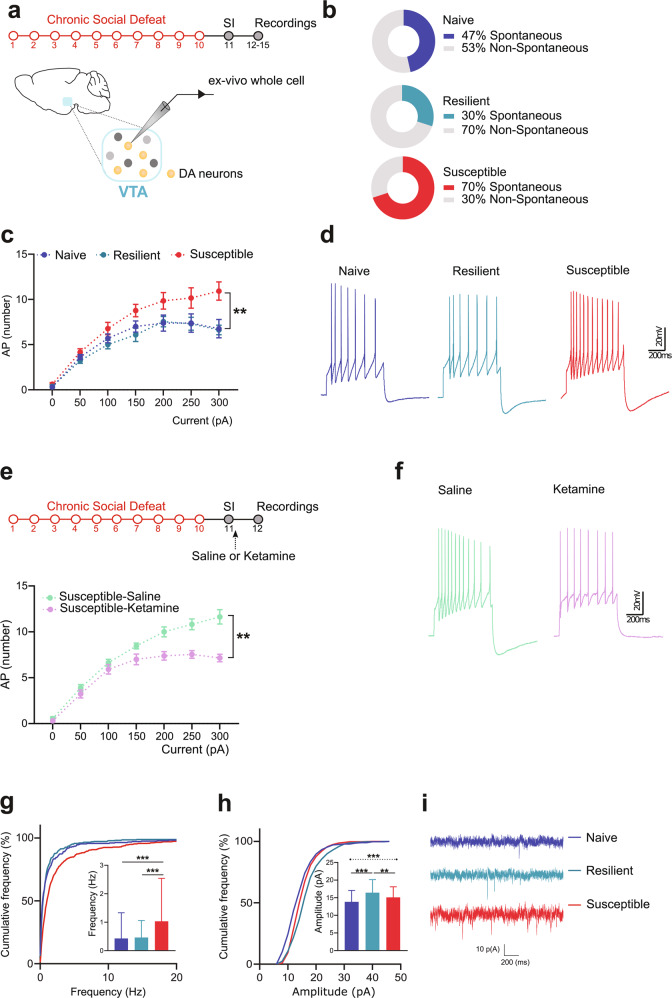
Chronic social defeat alters the firing rates and excitatory synaptic transmission of VTA DA neurons in females. **a** Schematic experimental timeline. **b** Percentage of cells with or without spontaneous firing for each condition (N/n: Naive = 13/4; Resilient = 13/3; Susceptible = 13/4). **c** VTA DA displayed increased excitability in susceptible mice. (N/n: Naive = 13/4; Resilient = 13/3; Susceptible = 13/4). **d** Representative voltage traces are shown. **e** Ketamine administration reverses the VTA DA cells hyperexcitability in susceptible mice. **f** Representative traces of recordings. **g** Graph plot and frequency distribution histogram depict significant increases in frequency of sEPSC in VTA DA neurons of susceptible mice. (N/n: Saline = 11/2; Ketamine = 13/2). **h** Graph plot and frequency distribution histogram show increased amplitude of sEPSC of VTA DA neurons in both susceptible and resilient groups. (N/n: Naive = 13/4; Resilient = 13/3; Susceptible = 13/4). **i** Representative traces of sEPSC. (N/n: Naive = 13/4; Resilient = 13/3; Susceptible = 13/4). Data are expressed as the mean ± SEM except for (**g**, **h**) where data are expressed as the median and interquartile range. N/n Number of cells/mice.

To determine the impact of social stress on VTA DA neurons, we next studied the changes in neuronal firing and spontaneous excitatory postsynaptic currents (sEPSC) using whole-cell patch clamp recordings ex-vivo (Fig. 2a). In naive mice, we found that 47% of VTA DA neurons showed spontaneous firing activity (Fig. 2b). Chronic social defeat triggered a robust increase in the number of neurons with spontaneous activity (70%) in susceptible mice, while the population of VTA DA neurons spontaneously active was low (30%) in resilient ones (Fig. 2b). Chronic social defeat produced a marked increase in excitability of VTA DA neurons in susceptible females, characterized by higher discharge frequency upon increasing depolarizing current injections (Fig. 2c, d, Repeated-measures two-way ANOVA, F (12, 216) = 2.85, *p* = 0.001; Tukey’s comparisons test: **p < 0.001). In contrast, this effect was absent in resilient mice for which the number of action potentials elicited were comparable to that of controls (Fig. 2c, d). Importantly, in susceptible mice, these changes in excitability were not associated with alterations in passive membrane properties, action potential and after-hyperpolarization potential shapes (Supplementary Fig. 2). To examine whether these cellular adaptations could be reversed by antidepressant treatment, we measured the excitability of VTA DA neurons in susceptible mice 24 h after receiving an acute injection of either saline or ketamine. As expected, saline-treated susceptible mice exhibited an increased excitability of VTA DA neurons (Fig. 1e, f). This effect was rescued following ketamine administration therefore identifying a cellular substrate for ketamine’s impact on the brain of female mice (Fig. 1e Repeated-measures two-way ANOVA: interaction experimental condition × current F (6, 132) = 8.971, *p* < 0.0001; Sidak’s comparisons test: **p < 0.001). To assess the impact of stress on glutamatergic transmission onto VTA DA neurons, we analyzed changes in the frequency and amplitude of sEPSC. Chronic social defeat caused a rightward shift in the cumulative probability plot for both frequency (Fig. 2g, i, Kruskal–Wallis *H* = 95.16, *P* < 0.0001, Dunn’s multiple comparisons test **p < 0.001, ***p < 0.0001) and amplitude (Fig. 2h, i, Kruskal–Wallis *H* = 48.34, *P* < 0.0001, Dunn’s multiple comparisons test ***p < 0.0001) in susceptible females, consistent with the reshuffling of VTA excitatory synapses at both pre- and post-synaptic sites. Resilient mice only showed increased amplitude of sEPSCs but with a greater magnitude than susceptible mice (Fig. 2g–i). These cellular adaptations were independent of the number of attacks or the estrus cycle as the percentage of animals in estrus or diestrus at testing were comparable in naive, resilient and susceptible mice (Supplementary Fig. 1a–c).

### Chronic social defeat induces a hypercholinergic state in both susceptible and resilient females

**Fig. 3:**
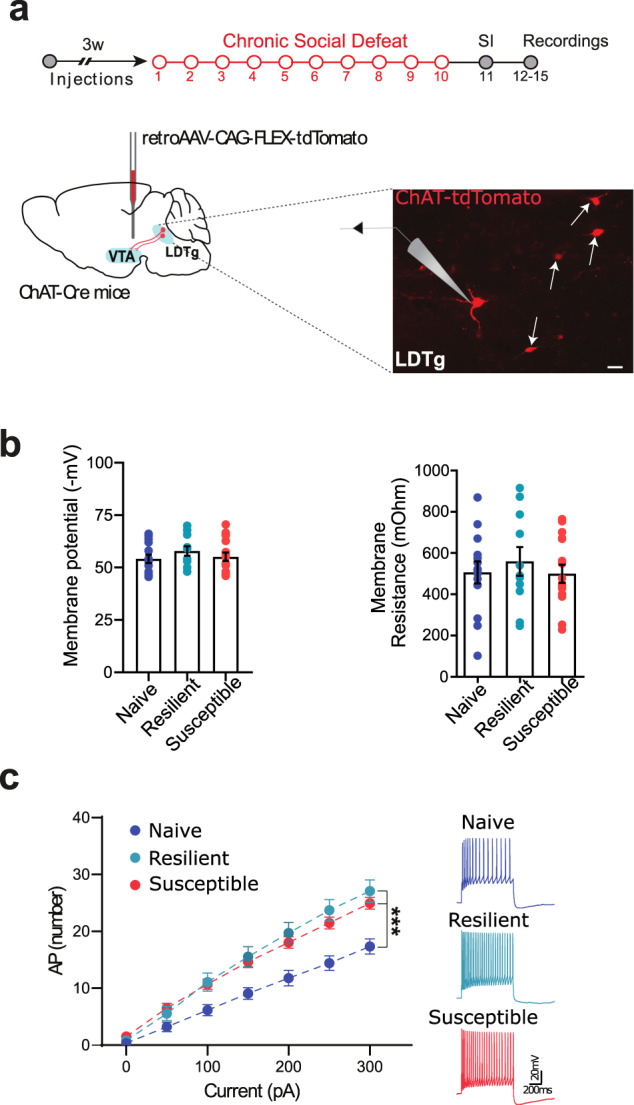
Chronic social defeat alters cholinergic signals in the VTA in females. **a** Schematic experimental timeline to assess the chronic social defeat effect on excitability of LDTg cholinergic cells projecting to the VTA. Three weeks before chronic social defeat, ChAT-Cre mice were injected with retro-AAV-CAG-FLEX-tdTomato bilaterally in the VTA. Confocal image shows tdTomato-tagged LDTg^→VTA^ cholinergic neurons. **b** No change in membrane potential and membrane resistance of LDTg^→VTA^ cholinergic neurons following chronic social defeat. **c** Cholinergic LDTg^→VTA^ neurons exhibited increased excitability profiles in both susceptible and resilient female mice. Representative voltage traces are shown (N/n: Naive = 14/2; Resilient = 11/2; Susceptible = 17/2).

Given the importance of cholinergic tone in shaping VTA DA neurons activity [23, 26, 30], we next examined whether chronic stress could affect the activity of cholinergic neurons in the LDTg that project to the VTA. To selectively record from LDTg cholinergic neurons inputs to the VTA, we injected ChATCre mice with a retroAAV-flex-tdTomato in the VTA (hereafter named ChAT^LDTg→VTA^ mice) (Fig. 3a). Chronic social defeat failed to alter resting membrane potential and resistance when compared to control (Fig. 3b). In contrast, chronic social defeat engendered a significant upward shift in the excitability profile of ChAT^LDTg→VTA^ neurons from mice that showed susceptible and resilient phenotypes (Fig. 3c, Repeated-measures two-way ANOVA, F (12, 234) = 8.48, *p* < 0.0001; Tukey’s comparisons test: ***p < 0.0001). This suggests an enhanced cholinergic tone within the VTA in both groups of defeated females.

### Differential sensitivity of nicotinic systems within the VTA of susceptible and resilient females

**Fig. 4:**
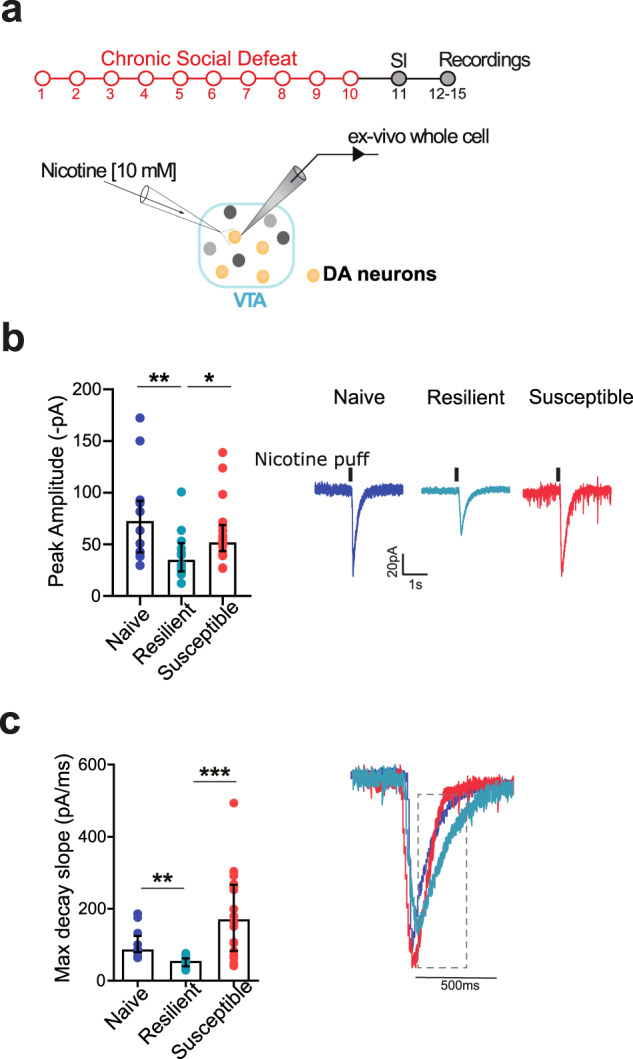
Chronic social defeat alters nicotine responses of VTA DA neurons in females. **a** Schematic experimental timeline to evaluate the response to nicotine of VTA DA cells in naive and defeated females. **b** Decreased nicotine-elicited currents from VTA DA neurons were found in resilient females. Representative recordings are shown. **c** Decreased decay constant of nicotine-elicited currents in resilient mice when compared to naive and susceptible (N/n Naive = 12/5; Resilient = 16/4; Susceptible = 19/9). Representative traces are shown.Representative traces are shown.

In light of this hypercholinergic tone, we next evaluated potential differences in post-synaptic signaling mediated by nAChRs expressed in VTA DA neurons that might participate in the increased dopaminergic activity observed after chronic social defeat. To that goal, we performed patch-clamp recordings of VTA DA neurons while delivering puffs of nicotine (Fig. 4a). Puffed nicotine elicited inward currents in all VTA DA neurons recorded. The peak amplitude was 78.66 ± 12.93 pA in naive mice and was comparable to that of females susceptible to chronic social defeat (61.64 ± 6.62 pA, Fig. 4b). In clear contrast, resilient mice exhibited nicotine-induced currents that were 49.62% smaller than controls (Kruskal-Wallis *H* = 10.53, *p* < 0.01, Dunn’s multiple comparisons test *p < 0.05; **p < 0.01). In addition, the analysis of the current kinetics revealed marked differences between the groups with resilient mice exhibiting smaller decay constant (Fig. 4c, Kruskal-Wallis *H* = 23.6 *p* < 0.0001, Dunn’s multiple comparisons test **p < 0.01; ***p < 0.001), likely to reflect alterations in the subunit composition of nAChRs expressed by VTA DA neurons. Overall, these data indicate that although cholinergic tone is increased in both susceptible and resilient mice, the latter adapts its post-synaptic response to acetylcholine perhaps as an adaptive mechanism.

### α7 nAChRs promote susceptibility to social stress in female mice

**Fig. 5:**
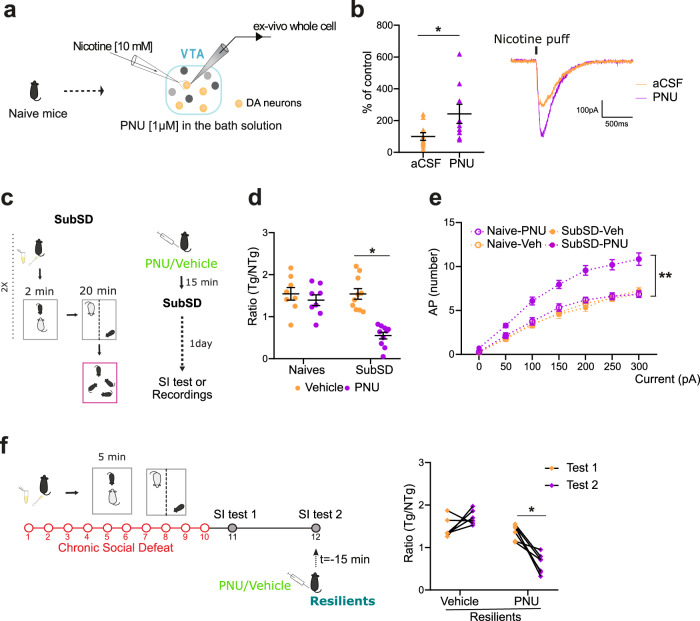
The modulation of α7-nAChRs triggers susceptibility to social stress in females. **a** Schematic of cellular actions of PNU-120596. **b** Bath application of PNU-120596 produces a significant increase in nicotine-elicited currents when compared to the bath application of vehicle alone. (N/n: vehicle = 10/3; PNU = 9/3). **c** Schematic of the subthreshold defeat procedure. **d** To evaluate the role of α7-nAchRs in the vulnerability to social stress, females received PNU-120596 (PNU) or vehicle injection paired with subthreshold social defeat and tested 1 day later in the SI test. PNU-injected females displayed significant social avoidance. (n/group: Naive-Vehicle = 8; Naive-PNU = 8; SubSD-Vehicle = 10, SubSD-PNU = 10). **e** VTA DA neurons displayed increased excitability only in female mice, which were submitted to subthreshold defeat stress paired with PNU-120596 injection. (N/n: Naive-Vehicle = 22/4; Naive-PNU = 21/4; SubSD-Vehicle = 19/4, SubSD-PNU = 25/4). **f** Experimental design to evaluate whether α7-nAchRs modulation can switch a resilient phenotype to a susceptible one. The graph depicts the social exploration of resilient mice during Test 1 (1 day following chronic social defeat) and Test 2 (1 day after T1 and 15 min following PNU administration). The positive modulation of α7-nAchRs promotes susceptibility to social stress in resilient females (n/group: Resilient-Vehicle = 5; Resilient-PNU = 7). Data are expressed as the mean ± SEM.

Of the different nAChRs subtypes present in the VTA, α7 nAChRs have been shown to be key for VTA DA neurons activity [24, 26, 33]. To interrogate their contribution, we employed PNU-120596, a positive modulator of α7-nAChRs [34]. We first validated ex-vivo that PNU-120596 could potentiate nicotine-elicited currents onto VTA DA neurons. Bath application of PNU-120596 onto VTA brain slices elicited a 100% increase of nicotine responses when compared to the control condition (Fig. 5a, b unpaired t-test, *t*(17) = 2.26, *p* < 0.05). Next, to test the potential involvement of α7-nAChRs in social stress-induced behavioral maladaptations, we used a protocol of subthreshold defeat. In this protocol, female mice were subjected twice to a mild 2-min encounter with the aggressor (Fig. 5c). In a first set of experiments, this protocol was combined with a single i.p. injection of vehicle or PNU-120596, a positive modulator of α7-nAChRs, and mice were tested 24 h after and compared to vehicle- and PNU120596-treated naive mice. While both naive groups as well as vehicle-treated mice exposed to subthreshold defeat showed high levels of social preference, mice exposed to subthreshold social defeat and PNU-120596 showed significantly lower interaction ratios (Fig. 5d Two-way ANOVA: significant interaction F (1, 32) = 11.99, *p* < 0.005; Sidak’s comparisons test: **p < 0.005). This increased sensitivity to stress enhanced by PNU-120596 was also noticeable at the cellular level. Indeed, only the combination of subthreshold defeat and PNU-120596 triggered an increased excitability of VTA DA neurons that was not observed in the other control conditions (Fig. 5e repeated-measures two-way ANOVA: F (18, 498) = 7.57, *P* < 0.0001). Next, we aimed at determining whether modulation of α7-nAChRs in mice previously exposed to a mild stressful experience could precipitate depressive-like behaviors. For this, we employed naive females as well as females that were exposed to subthreshold social defeat without pharmacological treatment. Twenty-four hours later, mice received either PNU-120596 or vehicle and rapidly tested for social interaction. Only the combination of PNU-120596 and subthreshold defeat favored the appearance of social aversion, while control groups showed normal pro-social behaviors (Supplementary Fig. 3 Two-way ANOVA: significant interaction F (1, 25) = 16.40, *p* < 0.0005; Sidak’s comparisons test: **p < 0.005). The effects of PNU-120596 were independent of the estrus cycle (Supplementary Fig. 1d, e). This set of data suggests that a mild stress exposure primes the reward system and that an immediate or delayed modulation of α7-nAChRs can promote the appearance of social avoidance in females.

Our previous results showed that resilient mice exhibit a diminished sensitivity of the nicotinic system in VTA DA neurons (Fig. 4). Because this could be a mechanism mediating resilience to stress, we next examined whether facilitating the activation of α7-nAChRs could reverse this phenotype and promote susceptibility. For this, a large group of female mice were submitted to chronic social defeat to isolate those showing resilience using the social interaction test (Fig. 5f, Test 1). Then resilient females were injected i.p. with PNU-120596 and were tested again 24 h later (Fig. 5f, Test 2). This pharmacological intervention engendered a marked susceptible phenotype in all animals tested (Repeated measure two-way ANOVA: significant interaction F (1, 11) = 25.72, *p* < 0.0005; Sidak’s comparisons test: *p < 0.005) regardless of the estrus cycle (Supplementary Fig. 1g). The injection of vehicle alone in resilient mice failed to decrease the ratio for social interaction, ruling out the possibility that the decrease observed in PNU-120596-treated resilient mice is due to habituation (Fig. 5f). This points to a key role of α7-nAChRs in favoring depressive-like symptoms.

## Discussion

Here we reveal in female mice significant dysregulation of VTA DA neurons activity that are associated with two behavioral hallmarks of depressive-like disorders, social aversion and anhedonia, and which behavioral abnormalities are reversed by acute ketamine injection. These cellular maladaptations are only observed in females susceptible to chronic social defeat, not in resilient mice, and can be reversed by a single ketamine injection. Although, both resilient and susceptible females exhibit a hypercholinergic tone in the VTA originating from the LDTg, we unraveled a diminished sensitivity of nAChRs located onto DA neurons only in resilient mice. Importantly, we identified that α7 nAChRs constitute a key molecular target that increases sensitivity to stress both at the behavioral and cellular levels. Therefore, these nAChRs subtypes favor the appearance of depressive-like symptoms. The present study thus contributes to understanding the neurobiological mechanisms that sustain stress-related disorders in females.

### The brain reward system and preclinical models of depression

The reward pathway has evolved to motivate animals to approach stimuli and engage in behaviors that are essential for survival. The study of DA neurons and the meaning of DA signals within this system has, and still generates strong debates in the scientific community [35–37]. In the recent years, it has become evident that DA neurons can both signal reward and aversion and therefore convey information about opposite motivational states [38–40]. These conclusions primarily arose from data gathered on male subjects. Here we show in females susceptible to chronic social defeat a marked increase in the firing activity of VTA DA neurons that is similar to what we, and other laboratories, have previously reported in males [15, 17, 18, 26, 30]. This indicates shared, if not common, cellular substrates for the appearance of depressive-like symptoms in both sexes. Changes in VTA DA neurons activity were also reported in female rats exposed to an unpredictable chronic mild stress (UCMS) procedure [41], a classical model known to induce depressive-like symptoms including anhedonia [42]. Indeed, a significant decrease in the population of active VTA DA neurons was measured in vivo in anaesthetized females previously submitted to UCMS, an effect that was fully reversed by ketamine and also observed in males [41]. Also, two other studies reported a decrease in the active population of VTA DA neurons and/or a decreased in the bursting and firing activities of VTA DA neurons in male mice exposed to UCMS [43, 44]. Overall, the two mostly employed preclinical models of depression, the UCMS and the chronic social defeat, produce decreases and increases of VTA DA neurons respectively, but these adaptations are independent of the sex. Comparing ex-vivo and in vivo recordings and performing correlation analyses in naive and socially defeated male mice, the group of Dr. Han concluded that increased firing rates were associated with the increase of bursting events [14]. This is likely to be the same in female mice but future studies employing in vivo recordings will be required to build a more comprehensive landscape of the adaptations occurring at VTA neurons in females, especially to disentangle the impact of social stress on bursting events, key to the appearance of depressive-like symptoms in males [15]. Although circulating gonadal hormones are potent modulators of brain functions [45], we found that susceptibility or resilience to chronic social defeat in females was not associated with differences in the estrus cycle, which is known to impact VTA DA neurons activity [46]. Hence, whether the underlying mechanisms leading to VTA DA neurons dysregulation is similar in male or female has yet to be determined in order to fully apprehend the complexity of this disease.

### Ketamine’s effects on VTA DA neurons activity

Although the US Food and Drug Administration approved only few years ago the use of ketamine in depression, it has been already more than two decades since ketamine, initially developed as an anesthetic drug, was first shown to have rapid therapeutic effects in patients with major depressive disorders [47]. Several clinical studies support antidepressant efficacy and safety of ketamine in both men and women [47–49]. This new discovery boosted the number of basic studies which sought to understand the neurobiological underpinnings of ketamine’s action [50]. Indeed, ketamine increased stress coping in antidepressant-predictive procedures such as the forced swim test [51], the novelty-suppressed feeding [52], and learned helplessness [53]. Ketamine also diminished depressive-like symptoms measured in mice exposed to UCMS or social defeat stress [54–56]. Yet, these experiments primarily used male mice. Here we show that an acute dose of ketamine in females susceptible to social defeat stress not only reverses social aversion but provides cellular benefits by fully restoring a normal firing activity of VTA DA neurons. Our results echo with a recent study demonstrating that in a learned helplessness procedure, repeated foot shocks induced aversive learning in males and females by impinging onto VTA DA neurons activity, measured using calcium imaging, and that this effect was sensitive to ketamine [57]. These results provide a strong impetus to further explore the role of DA signals in the physiopathology of stress-related disorders and to identify the cascade of events that explain ketamine’s impact onto the reward system.

### Cholinergic modulation of VTA DA neurons

The release of acetylcholine within the brain, either from local interneurons or long-ranged projecting ones, is key to modulate the activity of distinct neuronal networks and therefore impinges on a large behavioral repertoire, notably though its action onto nAChRs [33, 58, 59]. It is therefore evident that alterations in cholinergic signaling lead to pathological states [60]. Here we show that susceptibility to stress can be induced by the positive modulation of α7 nAChRs, an effect we previously reported in male mice [26]. Importantly, the same pharmacological manipulation abolished resiliency in females and triggered susceptibility to social stress. Hence, nAChRs may act as a switch to promote depressive-like states. The underlying cellular mechanisms for this resilient state is likely to implicate VTA DA neurons. Indeed, although resilient and susceptible females both exhibit increased LDTg cholinergic tone onto VTA DA neurons, only resilient females showed a poor reactivity of DA neurons to nicotine. This indicates that the integration of cholinergic signals differs between resilient and susceptible females, and that VTA DA neurons constitute a hub for susceptibility and resiliency to stress. This is further supported by the evidence that administration of PNU-120596 in mildly stressed mice increases the excitability of VTA DA neurons. Aside the contribution of α7 nAChRs, there are likely other contributing factors underlying resilience [17]. Indeed, we observed specific adaptations in resilient, but not susceptible, mice including more negative resting membrane potential and increased sEPSCs amplitude, which overall could affect VTA DA neurons activity in females.

What could be the mechanisms by which nAChRs affect VTA function in susceptible mice? Prolonged exposure to acetylcholine, as a result of increased LDTg cholinergic neurons activity, could affect the expression of nAChRs. As we report here a hypercholinergic states in both susceptible and resilient mice, the increased cholinergic tone could be a trigger but other unidentified actuators, likely in VTA DA neurons, should contribute to this process to explain the differential sensitivity of nicotinic systems. Excessive cholinergic tone has been proposed to contribute to the pathophysiology of major depression [61], however it is at present unknown whether molecular and/or functional adaptions in nAChRs play a role [62]. Our previous study conducted in males did not reveal any clear changes in nAChRs using radioligand binding assays performed on brain VTA slices or tissue fractionation [26]. Such changes may only occur in specific-neuronal subtypes given the heterogeneous distribution of VTA nAChRs [63] and therefore may be too subtle to detect on such preparations. In the VTA, α7 nAChRs are not associated with cholinergic synapses, suggesting more a role for cholinergic volume transmission [64]. Chronic stress could affect the local architecture of cholinergic circuits therefore altering the balance of nAChRs signaling. The testing of this hypothesis would require precise anatomical analyses to be solved in virally-tagged circuit and VTA cell populations. Overall, the study of nicotinic systems should help design better treatment in order to improve the pharmacopeia against depressive disorders.

Considering the scarce knowledge about pathophysiology of depression as well as biological underpinnings of resilience to social stress in females, our study provides valuable evidence revealing potential therapeutic targets for stress-related disorders in females.

## Supplementary information

Supplementary Figures and Legends
