# Supplementary material for: Nicotinic receptors promote susceptibility to social stress in female mice linked with neuroadaptations within VTA dopamine neurons
Source: Neuropsychopharmacology. 2022 Apr 22;47(9):1587–96. doi: 10.1038/s41386-022-01314-4 (PMC9283477; doi:10.1038/s41386-022-01314-4)
Supplement: Supplementary file 1 — Supplementary Figures and Legends [file 41386_2022_1314_MOESM1_ESM.pdf]

Title: Nicotinic receptors promote susceptibility to social stress in female mice linked with neuroadaptations within VTA dopamine neurons.

Vanesa ORTIZ*, Renan COSTA-CAMPOS*, Hugo FOFO, Sebastian P. FERNANDEZ and Jacques BARIK.

Supplementary Figures and Legends

Figure S1. The resilient and susceptible phenotypes do not depend on number of attacks or estrus cycle. a) Performance in the social interaction test for naive, resilient and susceptible female mice employed in electrophysiological studies (One-way ANOVA: effect of experimental condition $F(2,36) = 38.31$, $p < 0.0001$; Tukey's comparisons test: ***$p < 0.0001$; n/group: Naive = 12; Resilient = 12; Susceptible = 15). **b)** Number of attacks during defeat sessions (unpaired t-test, $t = 1.29$; $df = 25$; $p > 0.05$; n/group: Resilient = 12, Susceptible = 15). **c)** Estrus cycle in each condition at the day of recording. **d)** Estrus cycle of resilient female mice injected with PNU-120596 or vehicle before testing for social interaction. **e)** Estrus cycle of females subjected to subthreshold social defeat or not (naive) paired with PNU-120596 or vehicle and tested for social interaction (PNU pre-SubSD). **f)** Estrus cycle of females subjected to subthreshold social defeat or not (naive) paired with PNU-120596 or vehicle (PNU pre-SubSD) and used for electrophysiological recordings. **g)** Estrus cycle of females subjected to subthreshold social defeat or not (naive) and injected with PNU-120596 or vehicle just before testing for social interaction (PNU pre-SI) .

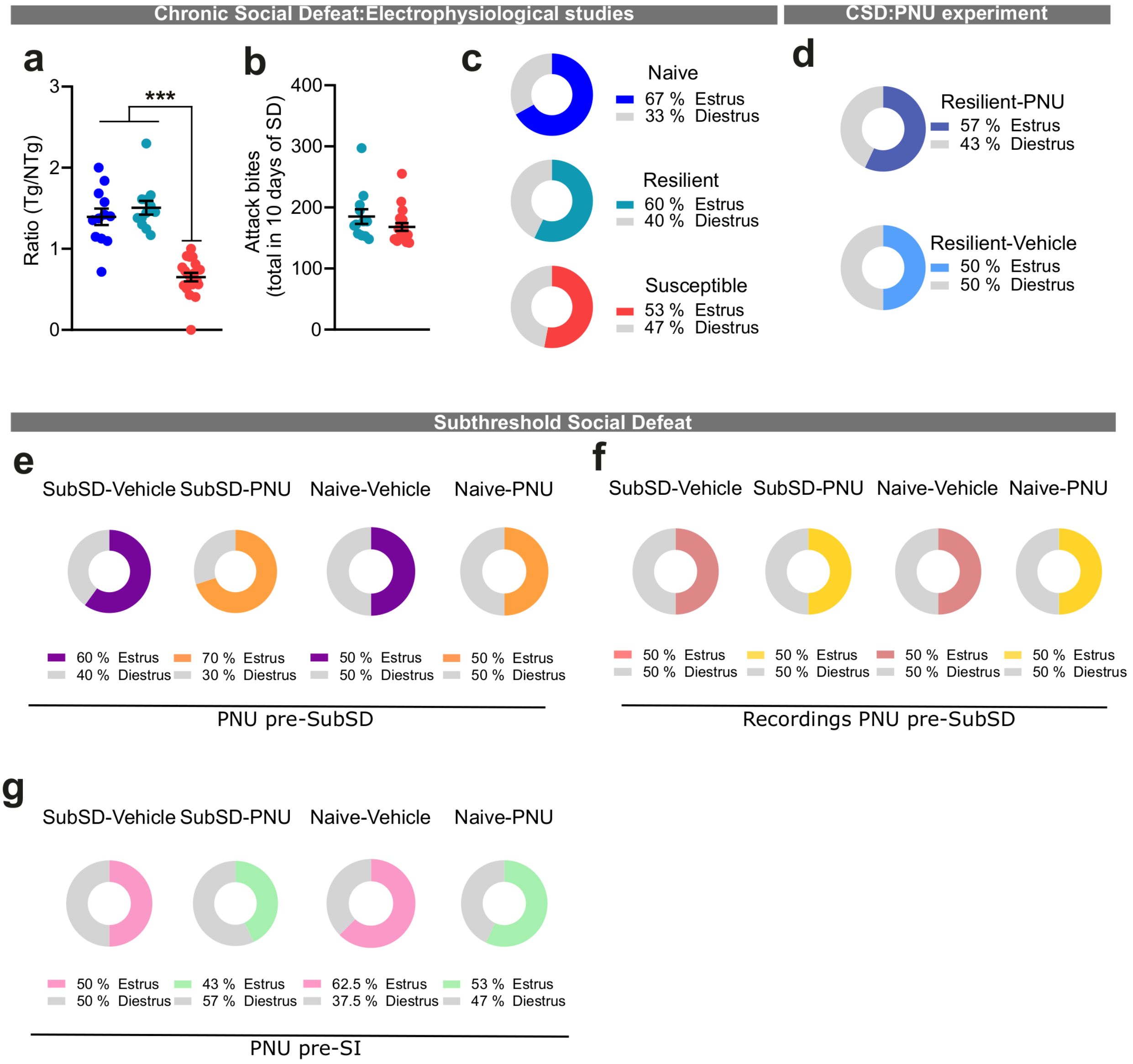

Figure S2. Intrinsic properties of VTA DA neurons in defeated females. a) Input resistance (One-way ANOVA: $F(2, 36) = 0.64$, $p > 0.05$). **b)** Membrane capacitance (One-way ANOVA: $F(2, 36) = 0.11$, $p > 0.05$). **c)** Resting membrane potential (One-way ANOVA: effect of experimental condition $F(2, 36) = 3.38$, $p < 0.05$). **d)** AP Threshold (One-way ANOVA: $F(2, 36) = 0.78$, $p > 0.05$). **e)** AP amplitude (One-way ANOVA: $F(2, 36) = 2.09$, $p > 0.05$). **f)** AP duration (Kruskal–Wallis $H = 0.11$, $p = 0.94$). **g)** AHP amplitude (One-way ANOVA: $F(2, 36) = 1.08$, $p > 0.05$). **h)** AHP time peak (Kruskal–Wallis $H = 3.36$, $p = 0.18$). N/n (number of cells/mice): Naive = 13/4; Resilient = 13/3; Susceptible = 13/4. AP: action potential. AHP: Afterhyperpolarization potential.

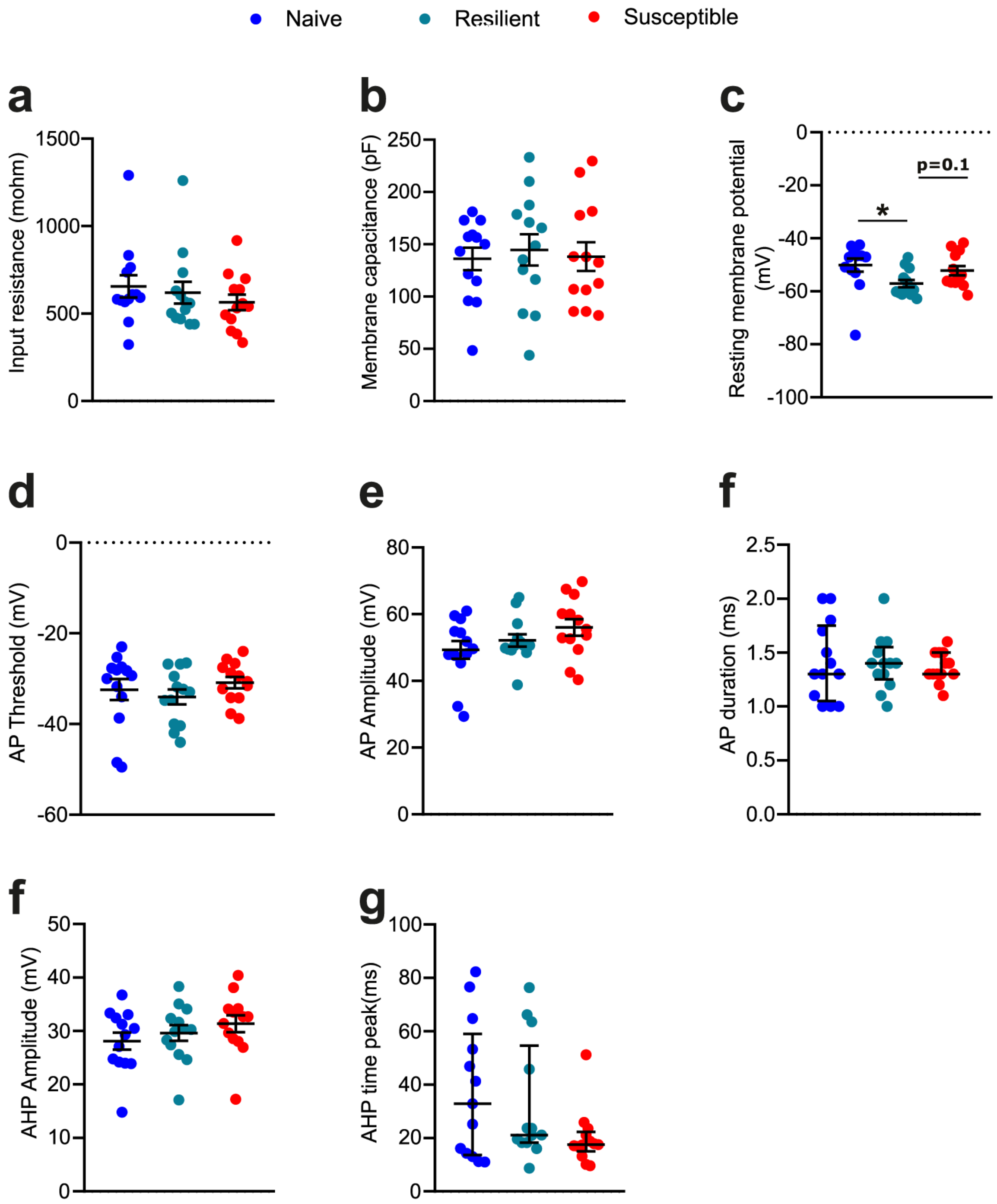

Figure S3. The modulation of α7-nAChRs triggers social avoidance in females previously primed with stress. Mice were subjected to subthreshold social defeat or not and tested 24h later. Fifteen minutes before SI testing, mice received vehicle or PNU. Social avoidance was only observed in mice primed with subthreshold social defeat and which received PNU.

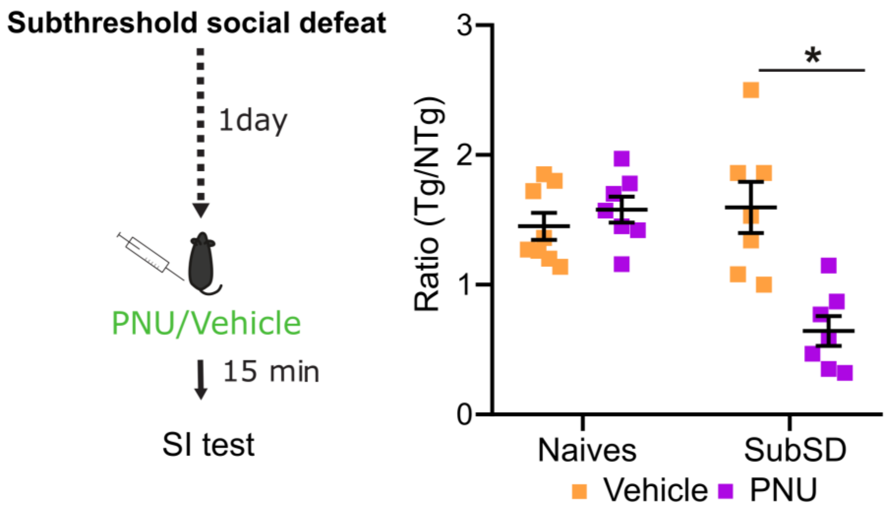

Supplementary table

Number of mice used per group for behavioral analyses

**Fig. 1c, d, e**	Naive	9	Resilient	8	Susceptible	8		
**Fig. 1f**	Naive-Saline	6	Naive-Ketamine	4	Susceptible-Saline	4	Susceptible-Ketamine	7
**Fig. 5b**	Naive-vehicle	8	Naive-PNU	8	Stress-vehicle	10	Stress-PNU	10
**Fig. 5f**	Resilient-vehicle	5	Resilient-PNU	7				
**Fig. S1**	Naive	12	Resilient	12	Susceptible	19		
**Fig. S3**	Naive-vehicle	8	Naive-PNU	7	Stress-vehicle	7	Stress-PNU	7

Number of cells/mice used per group used for electrophysiological recordings

**Fig. 2b,c,g,h,i**	Naive	13/4	Resilient	13/3	Susceptible	13/4		
**Fig. 2e**	Susceptible-Saline	11/2	Susceptible-Ketamine	13/2				
**Fig. 3b, c**	Naive	14/2	Resilient	11/2	Susceptible	17/2		
**Fig. 4b, c,**	Naive	12/5	Resilient	16/4	Susceptible	19/9		
**Fig. 5a**	aCSF	10/3	+PNU	9/3				
**Fig. 5e**	Naive-Vehicle	22/4	Naive-PNU	21/4	SubSD-Vehicle	19/4	SubSD-PNU	25/4
**Fig. S2**	Naive	13/4	Resilient	13/3	Susceptible	13/4		
